# The association between internet gaming disorder, depression, and anxiety among Palestinian university students who play internet games in a conflict-affected region

**DOI:** 10.3389/fpsyt.2026.1819488

**Published:** 2026-06-24

**Authors:** Muna Ahmead, Samah Abu Lail, Adnan Sarhan

**Affiliations:** 1 Faculty of Public Health, Al-Quds University, Jerusalem, Palestine; 2 An-najah National University, Department of Biomedical Sciences, Division of Public Health, Nablus, Palestine

**Keywords:** anxiety, depression, internet gaming disorder, loneliness, palestine, students, suicide ideation

## Abstract

**Background:**

Previous research has revealed that game addiction affects youth mental health. This study examined the association between internet gaming disorder (IGD), depression, anxiety, suicide ideation, and loneliness among university students who played internet games. The study utilized a cross-sectional design.

**Methods:**

A self-reported questionnaire was utilized to gather data, which included the Hospital Anxiety and Depression Scale, the Suicidal Behaviors Questionnaire-Revised, the Internet Gaming Disorder Scale (IGD), and the UCLA Loneliness Scale. A total of 1,228 students participated in the study. The participants completed an online survey via Enketo Express on the Kobo Toolbox platform. Data analysis involved the use of frequencies, percentages, the chi-square test, and multivariate regression analysis. The chi-square test was conducted to explore the relationships between internet gaming disorder and the study variables. Additionally, a multivariate regression analysis was carried out, with the results presented as adjusted odds ratios (AOR).

**Results:**

The results showed that 57.5% of university students were at a risk of IGD. The study found that females had higher odds of experiencing IGD than males (AOR:.668, p<.005). Additionally, participants with a household income of less than $350 were found to have lower odds of IGD compared to those with a family income exceeding $2500 (AOR: 0.427, p = 0.003). Furthermore, participants who reported anxiety (AOR: 1.806, p< 0.001), depression (AOR: 1.380, p= 0.038), and loneliness (AOR: 2.632, p< 0.001) were associated with increased odds of IGD in comparison to those without these conditions. Finally, participants who engaged in Internet gaming for 1-2hours (AOR: 3.093, p = 0.023), 3–4 hours (AOR: 3.786, p = 0.004) and over 4 hours (AOR:8.536, p=0.006) had higher odds of IGD compared to those who played for less than 1 hour daily.

**Conclusion:**

This study demonstrated significant associations between IGD, depression, anxiety, and loneliness among university students. University policymakers and mental health professionals have to increase students’ awareness of IGD and adopt early detection measures to identify students with significant risk factors for IGD.

## 1 Introduction

Students typically access the Internet through various devices for learning purposes in educational settings (1). The incorporation of online education into academic curricula has expanded students’ access to social media applications, including video games (2, 3). Participating in online gaming can effectively alleviate daily stressors (4). Further research has shown that gaming can boost self-esteem, help students build relationships, and promote a sense of social belonging (5, 6). Additionally, it supports cognitive development and enhances creative thinking (7). However, the widespread availability of smartphones, laptops, and mobile games has contributed to the rise of gaming disorder (8), which negatively impacts academic performance, mental health, and overall well-being among university students (1). Internet gaming disorder (IGD) is characterized by maladaptive usage of computer games, including both online and offline formats (9). The ICD-11 categorizes Gaming Disorder as “Disorders due to addictive behaviors.” Diagnosis requires a persistent gaming pattern marked by impaired control, prioritization of gaming over other activities, and continuation despite negative consequences. In general, a 12-month duration is necessary; however, in severe cases, the period may be reduced to six months (10). The Diagnostic and Statistical Manual of Mental Disorders (DSM-5) defines Internet Gaming Disorder (IGD) as a persistent pattern of excessive and prolonged engagement in Internet gaming. However, IGD was the only other behavioral addiction recommended for further research. This condition leads to various cognitive and behavioral symptoms, including a gradual loss of control over gaming, the development of tolerance, and withdrawal symptoms (11). Symptoms associated with IGD encompass an obsession with gaming, the development of tolerance, withdrawal symptoms, unsuccessful attempts to stop gaming, escapism from negative emotions, and jeopardizing important relationships or opportunities due to gaming (12). Globally, the prevalence of IGD ranges from 2.34% to 29.4% across various countries (13). Additionally, Gao et al. found that IGD is prevalent among both adolescents and young adults (9.9%), with adolescents showing a lower prevalence rate of 8.8% compared to young adults at 10.4% (14). The rising prevalence among young adults highlights the need for age-specific prevalence estimates and further investigation into the factors contributing to these variations (15).

University students are particularly vulnerable to IGD due to their extensive use of computers for academic, social, and recreational purposes (13). Furthermore, the stress students experience while adapting to a new and demanding academic environment increases their vulnerability to developing mental health problems (16), including anxiety, depression, suicidal ideation, and loneliness (13, 17–19). Research involving a representative sample of 1,531 adolescents and young adults has identified associations between IGD and increased symptoms of depression and anxiety (9). Additionally, other studies indicate that gamers often experience higher levels of depression compared to non-gamers (20). Furthermore, research suggests that depression may act as a risk factor for the onset of IGD and could influence its relationship with mental resilience and perceived stress (21). Also, internet gaming has emerged as risky online behavior and is used as a coping mechanism for students dealing with heightened levels of anxiety or stress as well as social, recreational, and escapism-related motivations. Snodgrass et al. highlight an association between anxiety and gaming addiction, noting that students frequently turn to gaming to escape real-world challenges, including academic pressure, interpersonal conflicts, and family tensions (22). This situation emphasizes the need for early identification of IGD, anxiety, and depression, particularly among at-risk groups such as university students, whose increased stress levels and demands may worsen these issues (23).

Furthermore, some students may use online platforms to alleviate feelings of loneliness and isolation (24). Loneliness is a subjective and uncomfortable experience that arises from a perceived gap between desired and actual relationships, characterized by feelings of isolation and detachment (25). Students who experience isolation often display maladaptive gaming habits as they use virtual connections to make up for inadequacy in their real-life relationships (26). Longitudinal research has revealed that loneliness is a predictor of gaming-related problems (27). In addition, research suggests that problematic gaming is associated with an increased risk of suicidal ideation (28, 29). According to Erevik et al., there is a moderate to substantial relationship between problematic video game use and suicidal thoughts (30). Additionally, a longitudinal study revealed that internet gaming disorder at age 25 significantly associates with suicidal ideation at age 28 (31).

The current research on IGD targets middle school and high school students, as well as the general population, with very few studies focusing on university students (12, 32, 33). To effectively support the mental health of students, it is essential for educational institutions, communities, and policymakers to evaluate the factors contributing to the increased prevalence of IGD (34). In Palestine, the pervasive use of technology such as computers, laptops, and smartphones among the younger generation may contribute to mental health problems. For example, a recent study revealed that 57.3% of students were at high risk of smartphone addiction (35). However, there is a lack of research examining IGD and its correlation with mental health problems. Therefore, this study aimed to assess the association between IGD and sociodemographic variables, depression, anxiety, suicidal ideation, and loneliness among university students who played internet games. Furthermore, it sought to determine the factors that were associated with IGD among these students. We hypothesized that Internet gaming disorder is significantly associated with sociodemographic variables, depression, anxiety, loneliness, and suicidal ideation among university students.

## 2 Methods and materials

### 2.1 Study design and sampling

In this study, male and female students aged 18 to 30 from 14 public universities in the West Bank were included. This group included both undergraduates and graduates who engaged in internet gaming. Students who did not play internet games were excluded. The study was conducted from April 23, 2025, to September 30, 2025. Using a 0.05 level of significance, a 95% confidence level, and a 3% accuracy, the study utilized SurveyMonkey to calculate a sample size of 1,067 university students as follows:


$$Sample\;size=\frac{\frac{z^{2}\times p\;\left(1-p\right)}{e^{2}}}{1+\left(\frac{z^{2}\times p\;\left(1-p\right)}{e^{2}N}\right)}$$

- N = population
- e = margin of error (percentage in decimal form)
- z = z-score* (how many standard deviations data is from the mean)
*95% confidence level is a 1.96 z-score

However, 1228 university students responded to the survey. Utilizing a non-probability sampling method that prioritizes accessibility within the target population, students were recruited through convenience sampling. On the Kobo Toolbox platform, participants completed an online survey using Enketo Express. The researchers subsequently distributed the survey through multiple various online channels, including student groups, Facebook, email, and WhatsApp.

### 2.2 Tools and measures

This study asked participants to complete a self-administered questionnaire consisting of five sections. The first section included socio-demographic data such as age, gender, marital status, monthly family income, faculty, year of study, location of residence, and parental education levels. In addition, this section contained questions about internet game usage, including the age at which participants began internet gaming, the duration of daily gameplay, emotional responses to online games, perceived pressure to engage in online gaming, and preferred types of games.

The second section incorporated the Internet Gaming Disorder Scale 9—Short Form (IGDS9-SF) (36), a concise tool for assessing the severity of IGD symptoms. The scale consists of nine elements. Each item is assessed using a 5-point Likert scale, ranging from (1 = never) to (5 = very often). The maximum and minimum potential scores on the IGDS9-SF are 45 and 9, respectively. Higher scores suggest increased severity of problematic internet gaming. The suggested cutoff score for the IGDS9-SF is 32, which is used by other studies (37–39). The instrument demonstrates a diagnostic accuracy of 96.1%, with a sensitivity of 98.0% and a specificity of 96.2%, based on a total of 29,020 cases (37). Research indicates that the instrument possesses significant advantages over most existing tools for IGD, along with strong psychometric properties (38, 40). Cronbach’s alpha (α) was found to be 0.87.

The third section involved the Hospital Anxiety and Depression Scale (HADS) (41), a 14-item instrument developed to evaluate the occurrence of anxiety and depression. The HADS constructs two instruments to differentiate between the two conditions: HADS-A for anxiety (seven items) and HADS-D for depression (seven items). Items are evaluated using a 4-point severity scale, where each question is scored from 0 (no impairment) to 3 (severe impairment), with three representing the maximum level of anxiety or depression. According to various studies, a case is deemed significant if the score on either scale is 11 or higher (41, 42). A score of 0–7 is regarded as within normal limits; 8–10 indicates mild depression, 11–14 corresponds to moderate depression, and 15–21 signifies severe depression. The Cronbach’s alpha coefficient was 0.80.

The fourth section included the Suicidal Behaviors Questionnaire—Revised (SBQ-R) (8), which consists of four questions. It aims to examine various aspects of suicidal behavior. The overall score ranges from 3 to 18. The SQB-R possesses a total score range from 3 to 18, with a score exceeding 7 signifying a greater risk of suicide ideation, which is used by other studies (43, 44).

The final section assessed loneliness using the UCLA Loneliness Scale (45), a 20-item questionnaire with scores ranging from 20 to 80, with higher scores indicating greater loneliness. Participants used a 4-point Likert scale (1=never, 2=rarely, 3=sometimes, and 4=always). The most common cut-off score for categorizing UCLA-LS-20 scores is >43, which was used by other studies (46, 47). Cronbach’s alpha (α) was 95.

A committee of five mental health professionals evaluated the instrument’s components to verify cultural appropriateness, and no revisions were recommended. The research team translated the scale into Arabic. This translation was then back-translated into English by a certified translator. In the pilot phase, the tool was distributed to 30 students to evaluate linguistic clarity. Both the original English questionnaire and its back-translated counterpart were reviewed to verify the accuracy of the translation.

### 2.3 Data analysis:

The data were analyzed using SPSS version 25 (IBM Corp., Chicago, IL, USA). The descriptive analysis for all research variables is presented as frequencies and percentages. The chi-square test was performed to examine the relationships between internet gaming disorder and study variables. A multivariate regression analysis was performed, and the findings were presented as adjusted odds ratios (AOR) with a 95% confidence interval.

In the multivariate logistic regression analysis, we incorporated variables anticipated to be associated with the study outcomes, irrespective of their significance in the univariate analysis. A p-value below 0.05 was considered significant. The absence of multicollinearity was confirmed through the use of variance inflation factors (with VIF values<5) and by assessing the stability of coefficients within the model. No variables were excluded due to collinearity.

### 2.4 Ethical consideration

All procedures employed in the present study adhered to the principles outlined in the Declaration of Helsinki. Al-Quds University Research Ethical Committee authorized the study (Ref No: 3/25R). This online survey was carried out anonymously. The study started with written information concerning its objectives and the manner in which the data will be employed. Participants provided informed consent for participation in this study by completing the questionnaire.

## 3 Results

### 3.1 Sociodemographic characteristics of the participants

In the present study, a total of 1,228 participants were recruited. The information presented in **Table 1** indicates that the majority of participants were female (71.4%), aged between 18 and 20, and unmarried. Additionally, 49.1% of the participants lived in villages, while 44.5% reported a family monthly income ranging from $651 to $1,600. Furthermore, 80.5% held a baccalaureate degree, and 35.5% were enrolled in arts faculties. Moreover, 56.3% of participants indicated that their fathers had 12 years of education or less, whereas 44.1% of their mothers had attained more than 12 years of education.

**Table 1:** Sociodemographic variables of the participants.

Characteristics		N	%
Age (years)	18-20	570	46.4%
	21-23	399	32.5%
	24-26	83	6.8%
	27-30	176	14.3%
Gender	Male	351	28.6%
	Female	877	71.4%
Marital Status	Single	1036	84.4%
	Not single	192	15.6%
Place of living	City	554	45.1%
	Village	603	49.1%
	Refugee camp	71	5.8%
Family monthly income	< 350 $	130	10.6%
	351- 650 $	240	19.5%
	651 -1600$	546	44.5%
	1601 - 2500$	191	15.6%
	>2500 $	121	9.9%
Level of education	Baccalaureate degree	989	80.5%
	Master, high diploma and Ph.D. degree	239	19.5%
Academic Year	First year	256	20.8%
	Second year	349	28.4%
	Third year	247	20.1%
	Fourth year	275	22.4%
	Fifth year	58	4.7%
	Sixth year	43	3.5%
Faculty	Faculty of Health Sciences	251	20.4%
	Faculties of Science	217	17.7%
	Faculties of Arts	436	35.5%
	Faculties of Engineering	130	10.6%
	Faculties of Business	194	15.8%
Father’s education Level:	≤ 12 years	691	56.3%
	>12 years	537	43.7%
Mother’s education Level	≤ 12 years	687	55.9%
	>12 years	541	44.1%

### 3.2 The risk of internet gaming disorder, suicide ideation, loneliness, anxiety and depression.

The data presented in **Table 2** indicate that 57.5% of participants were at risk of Internet Gaming Disorder (IGD). Furthermore, the findings revealed that 21.3% reported suicidal ideation, 88.2% experienced feelings of loneliness, 23.7% exhibited symptoms of depression, and 36.6% reported anxiety.

**Table 2:** The risk of internet gaming disorder, suicide ideation, loneliness, anxiety and depression.

Characteristics	Variables	F	%
Internet Game Addiction	< 32	522	42.5%
	≥32	706	57.5%
Suicidal ideation	<7	967	78.7%
	≥ 7	261	21.3%
loneliness	≤ 43	145	11.8%
	> 43	1083	88.2%
Anxiety	<11	778	63.4%
	≥11	450	36.6%
Depression	<11	937	76.3%
	≥11	291	23.7%

### 3.3 The association between internet game disorder and sociodemographic data

The findings in **Table 3** shows that there was a significant association between IGD and gender (p< 0.001).

**Table 3:** The association between internet game disorder and sociodemographic data.

Characteristics		Internet gaming disorder				
		< 32		≥32		
		N	%	N	%	P value
Age (years)	18-20	257	49.2%	313	44.3%	.286
	21-23	156	29.9%	243	34.4%	
	24-26	37	7.1%	46	6.5%	
	27-30	72	13.8%	104	14.7%	
Gender	Males	180	34.5%	171	24.2%	
	Females	342	65.5%	535	75.8%	<.001
Marital Status	Single	446	85.4%	590	83.6%	.372
	Not single	76	14.6%	116	16.4%	
Place of living	City	244	46.7%	310	43.9%	.434
	Village	252	48.3%	351	49.7%	
	Refugee camps	26	5.0%	45	6.4%	
Family monthly income	< 350 $	65	12.5%	65	9.2%	.212
	351- 650 $	98	18.8%	142	20.1%	
	651 – 1600$	232	44.4%	314	44.5%	
	1601 – 2500$	84	16.1%	107	15.2%	
	>2500 $	43	8.2%	78	11.0%	
Level of education	Undergraduates	426	81.6%	563	79.7%	.415
	Postgraduates	96	18.4%	143	20.3%	
Academic Year	First year	107	20.5%	149	21.1%	.159
	Second year	155	29.7%	194	27.5%	
	Third year	98	18.8%	149	21.1%	
	Fourth year	118	22.6%	157	22.2%	
	Fifth year	19	3.6%	39	5.5%	
	Sixth year	25	4.8%	18	2.5%	
Faculty	Faculty of Medicine	98	18.8%	153	21.7%	.224
	Faculty of Science	96	18.4%	121	17.1%	
	Faculty of Arts	178	34.1%	258	36.5%	
	Faculty of Engineering	66	12.6%	64	9.1%	
	Faculty of Business	84	16.1%	110	15.6%	
Father’s education Level:	≤ 12 years	281	53.8%	410	58.1%	.138
	>12 years	241	46.2%	296	41.9%	
Mother’s education Level	≤ 12 years	289	55.4%	398	56.4%	.725
	>12 years	233	44.6%	308	43.6%	

∗p< 0.05.

### 3.4 The association between internet gaming disorder and internet games use related variables, depression, anxiety, loneliness and suicide ideation

The findings presented in **Table 4** demonstrate significant associations between IGD and several factors, including the age at initial engagement in internet gaming; the average daily duration of gameplay; the type of device utilized for gaming; the emotional experiences during gameplay; the pressure to participate in gaming; and the negative impacts on academic achievement and mental health, as well as depression, anxiety, loneliness, and suicidal ideation, all of which are statistically significant with P< 0.001.

**Table 4:** The association between internet gaming disorder and internet games use related variables, depression, anxiety, loneliness and suicide ideation.

Characteristics		Internet gaming disorder				
		<32 (n=522)		≥32 (n=706)		
		N	%	N	%	P value
At which age did you begin playing Internet games? (years)	< 6	46	8.8	36	5.1	.001*
	7–10	170	32.6	213	30.2	
	11–15	196	37.5	240	34.0	
	16–18	57	10.9	112	15.9	
	> 18	53	10.2	105	14.9	
Number of hours spent playing Internet games daily	< 1 hour	206	39.5%	250	35.4	<0.001
	1–2 hours	186	35.6%	220	31.1	
	3–4 hours	80	15.3%	160	22.7	
	> 4 hours	50	9.6%	76	10.8	
What emotions did you have while playing internet games?	Positive feelings (e.g. calm, peace, and happiness.).	417	79.9	614	87.0	.001*
	Negative feelings (e.g. anger, tension and anxiety)	105	20.1	92	13.0	
Did you ever feel pressured to play Internet games?	Always	14	2.7	4	0.6	<0.001
	Sometimes	69	13.2	20	2.8	
	Rarely	104	19.9	69	9.8	
	Never	335	64.2	613	86.8	
What types of games did they particularly enjoy?	Action or adventure games (e.g., platformers, combat), sport games.	158	30.3	126	17.8	<0.001
	Puzzle or strategy games.	117	22.4	257	36.4	
	Role-playing games (RPGs)	31	5.9	21	3.0	
	Sports or racing games	45	8.6	48	6.8	
	Social or simulation games (e.g., building, farming, life simulations)	48	9.2	80	11.3	
	Online multiplayer games (e.g., Fortnite, Minecraft)	76	14.6	59	8.4	
	Other	47	9.0	115	16.3	
Suicidal ideation	<7	422	80.8%	545	77.2%	<0.001.
	≥ 7	100	19.2%	161	22.8%	
Loneliness	≤43	103	19.7%	42	5.9%	<0.001
	> 43	419	80.3%	664	94.1%	
Anxiety	<11	342	65.5%	436	61.8%	<0.001
	≥ 11	180	34.5%	270	38.2%	
Depression	<11	412	78.9%	525	74.4%	<0.001
	≥11	110	21.1%	181	25.6%	

∗p< 0.05.

### 3.5 Multivariate regression for internet gaming disorder

The multivariate logistic regression analysis presented in **Table 5** indicated that females were more at risk of IGD compared to males (AOR: 0.668, p< 0.005). Additionally, participants with a family income of less than $350 per month were at lower risk of IGD than those with a family income exceeding $2,500 (AOR: 0.427, p = 0.003). Those experiencing anxiety (AOR: 1.806, p< 0.001), depression (AOR: 1.380, p = 0.038), and loneliness (AOR: 2.632, p< 0.001) were at a risk of IGD in comparison to individuals without these conditions. Furthermore, participants who engaged in online gaming for 1–2 hours (AOR: 3.093, p= 0.023), 3–4 hours (AOR: 3.786, p = 0.004), and more than 4 hours (AOR: 8.536, p = 0.006) were also at a higher risk of IGD than those who played for less than 1 hour daily.

**Table 5:** Multivariate regression for internet gaming disorder.

Characteristics		Internet gaming disorder		Adjusted analysis 95% CI, AOR	
		Sig.	AOR	Lower	Upper
Gender	Males	.005	.668	.503	.887
	Females	Reference			
Family income per month	< 350 $	.003	.427	.243	.749
	351- 650 $	.222	.730	.440	1.210
	651 -1600$	.271	.774	.491	1.221
	1601-2500$	.159	.688	.408	1.159
	>2500 $	Reference			
Anxiety	≥11	<.001	1.806	1.371	2.378
	<11	Reference			
Depression	≥11	.038	1.380	1.019	1.869
	<11	Reference			
Loneliness	> 43	<.001	2.632	1.725	4.017
	≤ 43	Reference			
The daily duration of time spent playing electronic games	1–2 hours	.023	3.093	1.166	8.210
	3–4 hours	.004	3.786	1.517	9.453
	> 4 hours	.006	8.536	3.461	21.052
	< 1 hour	Reference			

AOR, Adjusted Odds Ration; 95% CI, 95% Confidence Interval; Sig., Significance level (p<0.05). The multivariate logistic regression model includes all study variables.

## 4 Discussion

The findings of the present study revealed that 57.5% of participants were at a risk of Internet Gaming Disorder (IGD), which is considered higher than what prior research reported in the literature review (34, 48–50). Several factors may contribute to this finding. The IGDS9-SF threshold of ≥32 has not been thoroughly examined in Palestinian communities. The ≥32 criterion, when adapted for a different cultural context, may exhibit increased sensitivity and decreased specificity in Palestinian samples, resulting in a higher rate of IGD than would be identified using a clinically validated diagnostic interview. Future research involving Palestinian university populations may employ established clinical diagnostic instruments, such as structured clinical interviews (e.g., SCID-5) and clinician-administered assessments for IGD. Additionally, Palestinian university students face unique sociopolitical stressors, including ongoing political conflict, restricted mobility, financial challenges, limited recreational opportunities, and increased societal stress, which may significantly increase their vulnerability to problematic gaming (51, 52). Moreover, our sample comprised university students—a group that is notably youthful and technologically competent compared to the general population. The literature consistently links such demographic characteristics to a higher rate of Internet Gaming Disorder (IGD) (37, 53). Additionally, although our sampling strategy aimed to ensure representativeness within the university context, it may have inadvertently overrepresented students who engage more heavily in gaming, particularly due to recruitment through digital platforms.

Our findings identified multiple factors that were associated with increased the risk of IGD among the participants in our study. The results indicated that females were at a risk of IGD compared to males, which is inconsistent with previous studies (17, 54–56). A meta-analysis highlighted gender differences in addiction pathways, suggesting that males are more likely to experience IGD, while females are more susceptible to social media addiction (56). It has been suggested that the competitive elements, socially sanctioned aggression, and sexualized content of games can make them more appealing to males than to females, thereby increasing the risk of Internet Gaming Disorder (56, 57). Research indicates that males tend to exhibit higher levels of aggression, while women are more inclined toward social interaction, escapism, coping strategies, fantasizing, and participation in leisure activities (58). Initially and throughout the maintenance phase of IGD, males demonstrate greater impulsivity and reward-seeking behaviors, whereas females experience more pronounced emotional dysregulation (59). Consequently, it is essential to increase female students’ awareness and education regarding compulsive online gaming.

Furthermore, our findings revealed that participants with a family income below $350 per month were at a risk of IGD compared to those with a family income exceeding $2,500, which aligns with the results of other studies (60). This finding contrasts with other research suggesting that higher family income is associated with a reduced risk of IGD (61–63). Additionally, research has shown that lower monthly household income is positively associated with IGD (33, 34) and that diminished socioeconomic status is a significant risk factor for IGD. Studies suggest that adolescents from low-income backgrounds who engage in gaming as a form of escapism are particularly vulnerable to gaming addiction (63). The authors proposed that these students may experience heightened levels of stress and self-criticism, which could lead them to use gaming as a coping mechanism for stress relief (34). One possible explanation for our findings is that most internet games are not freely accessible in Palestine, and students from low socioeconomic backgrounds may lack the financial means to purchase or play them. As a result, their usage of these games is lower compared to students from high-income families who can afford them. Recognizing the economic status of students at risk for IGD is crucial for developing targeted intervention strategies. Therefore, it is necessary to focus on students from high-income families and to raise their awareness of the potential hazards associated with these activities in order to decrease their use of these games.

Further, the current study revealed that participants experiencing anxiety or depression were at a risk of IGD compared to those without these conditions. This finding aligns with prior research (34, 64–67) but contrasts with other studies (68). Approximately one in three individuals with IGD also experience depression (69). Several studies have identified depression and anxiety as consequences of problematic gaming (65, 70), while others have shown a bidirectional relationship between depression and gaming disorder (71). Falcione et al. found that depressive symptoms are significantly associated with subsequent gaming disorder, with anxiety being the next most influential factor (72). Research indicates that moderate engagement in gameplay may relieve symptoms of anxiety, tension, and depression, as well as enhance cognitive function (73) and offer psychological benefits (74). Nonetheless, participation in gaming activities may facilitate addiction by providing temporary relief from depression and generating feelings of happiness, similar to the effects that addictive substances exert on the brain’s reward system (75, 76). One possible explanation for the current finding is the high prevalence of mental health problems among students at Palestinian universities. Research has shown that individuals may experience depression in the real world and turn to virtual environments to alleviate their negative moods (77). However, excessive use of these virtual devices can exacerbate depressive symptoms (78). Recent studies suggest that Palestinian universities are at a high risk of depression, anxiety, and post-traumatic stress disorder, largely due to ongoing political violence and difficult social and economic conditions in Palestine (51, 52, 79). However, this could be a cumulative response to continuous exposure to political pressures, rather than a reflection of individual dispositions. Our findings may underscore the need for addressing gaming behaviors and mental problems among university students.

Moreover, the current study found that individuals who reported feelings of loneliness were at a risk of IGD compared to those who did not express such emotions, which aligns with previous research (64, 80). Research on the relationship between video gaming and loneliness has produced inconsistent results (81). Some studies indicate positive association, such as a reduction in feelings of loneliness among university students (82, 83). Williams (2018) argues that video games have a dualistic impact on psychosocial aspects, suggesting that playing online games can both enhance and diminish important social connections (84). Attasara et al. found that 71.5% of participants used gaming to “alleviate loneliness,” while 92.4% indicated “relaxation” as their primary motivation (85). Lonely persons may find pleasure in playing video games online, according to the theory of compensating Internet use. This benefit is due to its capacity to reduce negative emotions and provide unsatisfied social needs (86). According to Attasara et al., cyber games function as a digital platform for teenagers to meet new people, feel valued, and alleviate their loneliness (85). Those who spend too much time gaming online may feel lonely because they talk more with their online friends than with their real-life peers. People spend less time engaging with one another in person, making it more difficult to meet new people and perhaps affecting present relationships (87). Consequently, students may increase social engagement to assist them in mitigating feelings of loneliness and diminishing their dependence on digital platforms for emotional expression and the formation of social connections with strange individuals.

Also, the findings of the current study showed that individuals who played on the internet for 1–2 hours, 3–4 hours, or more than 4 hours were at a greater risk of IGD than those who played for less than 1 hour every day. This conclusion is consistent with previous studies (88–90) but differs from previous findings (34, 66, 91). A previous study has shown that those who play digital games for more than five hours per day have significantly a higher risk of addiction ratings than those who play for less than one hour (88). According to Kuss and Griffiths, excessive interaction with digital games is associated with addiction and has a negative influence on social connections. As a result, when a person devotes a substantial amount of time to gaming, they may gradually acquire an addiction, making it increasingly difficult for them to discontinue playing (89). This addiction may be associated with emotional instability, impulsive decision-making, and decreased focus, which may heighten the likelihood of impulsive behaviors (90).

Finally, the current study’s results did not demonstrate a statistically significant association between suicidal ideation and IGD, which aligns with prior research (91) but contrasts with the findings of other studies (8, 92). Junus et al. conducted a systematic review revealing a significant association between IGD and suicidal ideation among younger populations. Excessive gaming may exacerbate feelings of loneliness and despair, potentially leading to an increase in suicidal ideation, particularly within high-stress disciplines (92). There are many potential explanations for the current study findings; the Palestinian Ministry of Telecommunication and Digital Economy regulates the categories of games accessible via the internet. They prevent games that promote suicidal behavior, thereby safeguarding students from exposure to hazardous games such as Roblox (93). Furthermore, our study found that the majority of students (75.8%) engaged with action games, puzzles, role-playing games, sports or racing games, and social or simulation games. Therefore, they exhibited a lower risk of experiencing suicidal ideation associated with IGD. Additional research is necessary to investigate the underlying causes of suicidal ideation within this age group.

This study has some limitations. This study employs a cross-sectional design, which limits its ability to establish causality or identify long-term trends. Additionally, the use of self-administered questionnaires may introduce biases, as participants might underreport or over report their symptoms. The reliance on convenience sampling increases the likelihood of sample bias, as students with severe intellectual disabilities, severe depression, anxiety, or Internet Gaming Disorder (IGD) may either choose not to participate or be disproportionately represented due to their heightened interest in the survey. Another limitation of this study is the use of established cutoff scores for categorizing continuous scale scores. While this approach aligns with the prevalence-oriented goals of the research, this approach may reduce variability, statistical power, and interpretive precision. Despite these limitations, this research represents the preliminary nationwide study to assess Internet Gaming Disorder (IGD) among young adults in Palestine.

### 4.1 Implication for practice

Our study found a high risk of IGD symptoms among university students, indicating that it is a significant health issue in Palestine. Providing early identification, treatment, and support can significantly improve results for university students, allowing for academic and emotional growth. The findings emphasized the need for adopting psychological and therapeutic treatments for IGD, depression, anxiety, and loneliness to reduce the likelihood of academic and personal challenges while also promoting overall well-being among university students. Universities should establish targeted mental health support programs for students, particularly female students, to assist them in managing the psychological stressors associated with elevated levels of IGD. Educational initiatives should be designed to promote awareness of the dangers of excessive gaming, encouraging students to engage in moderate gaming activities that do not jeopardize their academic achievement or personal well-being.

Establishing accessible counseling and support networks on campus can facilitate students in managing IGD by providing essential resources and competencies for effective mental health management. Furthermore, creating a secure and supportive academic environment, along with fostering positive relationships between students and teachers, is crucial for reducing feelings of loneliness. Additional research is necessary to investigate the risk factors associated with IGD among university students. Further research may provide a more comprehensive understanding of gaming behaviors and their correlation with suicidal ideation. Furthermore, adopting a longitudinal design would enable a more thorough comprehension of the long-term effects of gaming and its potential contribution to the development of IGD and mental health conditions. Furthermore, employing a broader range of sampling methods would enhance the capture of a more diverse array of experiences, taking into account cultural and familial differences that may influence gaming behavior.

## 5 Conclusion

This study emphasizes the significant relationship between Internet Gaming Disorder (IGD), depression, anxiety, and loneliness in university students. University policymakers and mental health professionals should implement early detection tools to identify students with significant risk factors for IGD. By emphasizing preventive techniques for IGD and associated risk factors, universities may provide students with the means to cultivate healthy coping mechanisms. Promoting gaming alternatives can substantially improve general well-being and mental health of these students.

## Data Availability

The original contributions presented in the study are included in the article/supplementary material. Further inquiries can be directed to the corresponding author.
